# QT interval evaluation associated with the use of hydroxychloroquine with combined use of azithromycin among hospitalised children positive for coronavirus disease 2019

**DOI:** 10.1017/S1047951120002425

**Published:** 2020-07-20

**Authors:** Tunc Tuncer, Mehmet Karaci, Aysun Boga, Hilal Durmaz, Sirin Guven

**Affiliations:** Department of Pediatrics, Prof. Dr. Ilhan Varank Training and Research Hospital, Istanbul, Turkey

**Keywords:** coronavirus disease 2019, hydroxychloroquine, azithromycin, QT interval

## Abstract

**Introduction and aim:**

Hydroxychloroquine alone or in combination with azithromycin has been increasingly used for patients with coronavirus disease 2019, in both children and adults. Drugs are generally well tolerated in clinical practice; however, both can cause corrected QT prolongation. We aimed to report our experience of QT interval evaluation associated with the use of hydroxychloroquine with concurrent azithromycin among children testing positive for coronavirus disease 2019.

**Methods:**

Our single-centre; retrospective, study evaluated children with coronavirus disease 2019 disease admitted to the Pediatric Department at Sancaktepe Training and Research Hospital Istanbul, Turkey from 10 March, 2020 to 10 April, 2020. The data including demographics, clinical symptoms, co-morbid diseases, laboratory, radiological findings as well as electrocardiographs of the patients were obtained from our records. Electrocardiograms were evaluated before, one day after and at the termination of the treatment.

**Results:**

21 patients aged 9 to 18 years were evaluated. The median age was 170 months (range 112–214), 51.1% of them were girls and 48.9% were boys. Their laboratory results did not reveal any abnormalities. None of them needed intensive care. We did not detect QT prolongation during or at the termination of the treatment.

**Conclusion:**

We did not detect QT prolongation during or at the termination of the treatment in our patients due to the fact that they were not severely affected by the disease. Patients were treated in our inpatient clinic and none of them required intensive care. Laboratory results were also insignificant. Furthermore, they did not need other medications.

The novel coronavirus disease 2019, caused by severe acute respiratory syndrome coronavirus 2, is continuing to spread. Children and newborns generally are asymptomatic or can present with atypical symptoms, such as low grade fever, vomiting, diarrhoea, mild fatigue, and cough without any signs of pneumonia or shortness of breath. Due to these subdued symptoms, patients can end up visiting outpatient healthcare centres multiple times before becoming a confirmed case, which contributes to the spread of the virus.^1–3^

Studies in patients with mild to moderate coronavirus disease 2019 symptoms have suggested benefits of hydroxychloroquine alone or in combination with azithromycin against severe acute respiratory syndrome coronavirus 2 and raised hope for treating the disease.^4^ Although hydroxychloroquine and azithromycin are generally well-tolerated medications used in clinical practice, both can cause corrected QT prolongation.^5,6^ However, most of the studies and reviews are based on adult data so that studies are needed to evaluate the effects of these medications on children’s cardiovascular system.

In this study, we aimed to characterise the risk and degree of QT prolongation in children with coronavirus disease 2019 in association with their usage of hydroxychloroquine with or without concomitant azithromycin.

## Materials and methods

This was a single-centre retrospective study evaluating children with coronavirus disease 2019 who were hospitalised at the Pediatric Department at Sancaktepe Training and Research Hospital Istanbul, Turkey. The data including demographics, clinical symptoms, severity of disease, co-morbid diseases, laboratory and radiological findings (chest X-ray and CT imaging) as well as electrocardiograms of the patients were obtained retrospectively from hospital records. We included patients admitted between 10 March, and 10 May, 2020, who received 5 days of hydroxychloroquine with or without concomitant azithromycin while inpatients and at least one positive coronavirus disease 2019 nasopharyngeal polymerase chain reaction test. Electrocardiograms were manually evaluated before, one day after and at the termination of the treatment which were compared consequently. The standard regimen was 400 mg of hydroxychloroquine twice on day 1, then 400 mg daily on days 2 through 5. Azithromycin was given once 500 mg at the first day and 250 mg once a daily for the other four days. Electrocardiograms were manually evaluated by our clinic’s paediatric cardiologist to calculate corrected QT intervals using the Bazett formula and so-called excess correction method for QRS values greater than 120 milliseconds. Standard 12-lead electrocardiogram tracing at 25 mm/s paper speed at 10 mm/mV amplitude was used for accurate measurement of QT interval duration. QT interval was determined as a mean value derived from at least 4 cardiac cycles and measured from the beginning of the earliest onset of the QRS complex to the end of the T wave. The QT measurement was made in leads II and V_5_ or V_6_, with the longest value being used. If there were T and U waves that are close together, we identified the end of the T wave when it is descending limb returns to the TP segment (TP) baseline that it is not followed by a U wave or it is distinct from the following U wave. When T-wave deflections of equal or near-equal amplitude result in a biphasic T wave, the QT interval is measured to the time of final return to baseline. End points of interest were changes in corrected QT higher than 60 milliseconds between consecutive electrocardiograms, development of prolonged corrected QT interval to 500 milliseconds or more, and documented adverse drug events, in the cohort receiving only hydroxychloroquine and hydroxychloroquine plus azithromycin.

### Statistical analysis

Nominal data were described using proportions. Normally distributed discrete data were described with means and medians; and minimums and maximums were used to represent data that were not normally distributed. Statistical analyses were performed using SPSS version 25.0 (IBM, Armonk, New York, USA).

## Results

A total of 21 patients suspected for coronavirus disease 2019 infection aged 9 to 18 years were evaluated. The median age was 170 months (range 112–214). % 51, 1 (n = 12) of them were girls and % 48, 9 (n = 9) were boys. None of patients were critically ill at the time of testing. None of patients had cardiovascular co-morbidity, and they were not taking corrected QT prolonging medications. Two of them were asthmatic and one of them had epilepsy. Coughing was the most common symptom encountered. 16 (76.1%) of the patients had cough as a symptom with other symptoms like fever, loss of smell, malaise, diarrhoea and vomiting. Their laboratory test results at admission were insignificant and are depicted in Table 1. However, all were tested positive for coronavirus disease 2019 nasopharyngeal polymerase chain reaction test. Fifteen patients had ground-glass opacities and air space consolidations on their chest X-rays. Twelve of them had also ground-glass opacities on their CT images. Two patients received hydroxychloroquine, and 19 (90.4 %) received hydroxychloroquine plus azithromycin. The baseline corrected QT values, values one day after the treatment, and at the termination of the treatment are all shown in the table. After the onset of treatment, patients had a mean Δ corrected QT of 0.8 milliseconds compared with the baseline electrocardiograms, whereas after the termination of the treatment mean Δ corrected QT was 4.9 milliseconds. One of the patients receiving hydroxychloroquine plus azithromycin developed prolonged corrected QT of 55 milliseconds more than the previous electrocardiogram. Patient’s baseline corrected QT was 361 milliseconds which turned out to be 416 milliseconds after the treatment. However, the third electrocardiogram revealed a corrected QT of 384 milliseconds. As the ΔQTc was under 60 milliseconds, we did not change the course of the treatment of the patient.

**Table 1.** Demographics, laboratory, radiological findings and corrected QT values of the patients

Characteristics	Total (n = 21)	Hydroxychloroquine (n = 2)	Hydroxychloroquine and azithromycin (n = 19)
Age (month) mean (median, min, max)	170 (170, 112, 214)	183 (193, 159, 206)	169 (170, 112, 214)
Female (%)	12 (57.1)	1 (50)	11 (52.3)
BMI mean (median, min, max)	23.7 (22.9, 17.1, 35.5)	27.5 (27.25, 19.5, 35)	23.3 (22.9, 17.1, 35.5)
Baseline laboratory, values, median, (min, max)			
White blood cell count, cells/μL	7400 (3500, 17100)	8550 (5500, 11600)	7400 (3500, 17100)
Neutrophil/lymphocytes, mean	2.7	2	2,7
Platelet × 1000	262 (159, 407)	308 (278, 339)	255 (159, 407)
Haemoglobin, g/dl	13.1 (10.6,16,3)	13.8 (12.2, 15.5)	13.1 (10.6, 16,3)
C-reactive protein, mg/dl	1.04 (0, 0.9)	0.37 (0.01, 0.73)	1.12 (0, 0. 9)
Serum creatinine, mg/dl	0.66 (0.57, 1.02)	0.66 (0.6, 0.73)	0.66 (0.57, 1.02)
D-dimer, μg/mL mean, (min, max)	0.1 (0, 0.73)	0 (0, 0, 0)	0.11 (0, 0.73)
Troponin, pg/ml	0.7 (0, 16, 9)	0.9 (0.6, 1.2)	1.66 (0, 16.9)
Magnesium, mg/dl	1.98 (1.7, 2.31)	2.08 (1.88, 2.28)	1.98 (1.7, 2.31)
Potassium, mmol/l	4.2 (3.8, 5.2)	4.3 (4.1, 4.5)	4.2 (3.8, 5.2)
Calcium, mg/dl	8.9 (7.5, 10)	8.5 (8.5, 8.5)	9 (7.5, 10.2)
Radiographic findings			
Chest X-ray findings of pneumonia (%)	15 (71.4)	2 (100)	13 (68.4)
CT findings of pneumonia (%)	12 (57.1)	2 (100)	12 (52.6)
Electrocardiogram (ms), mean, median, min, max			
Baseline QTc	408 (410, 357, 486)	440 (440, 395,486)	405 (410, 357, 433)
Post-treatment QTc	396 (400, 356, 450)	415 (415, 404, 426)	394 (395, 356, 450)
ΔQTc pre- versus post-treatment	-8 (-7, -84, 51)	-35 (-35, -84, 13)	-5 (-7, -52, 51)
ΔQTc pre-treatment versus one day after the treatment	0.6 (-1, -86, 55)	-46 (-46, -86, -7)	5.6 (4, -35, 55)

BMI calculated as weight in kilograms divided by height in metres squared

Abbreviations: BMI = body mass index; ΔQTc = change in corrected QT interval

**Figure 1.**
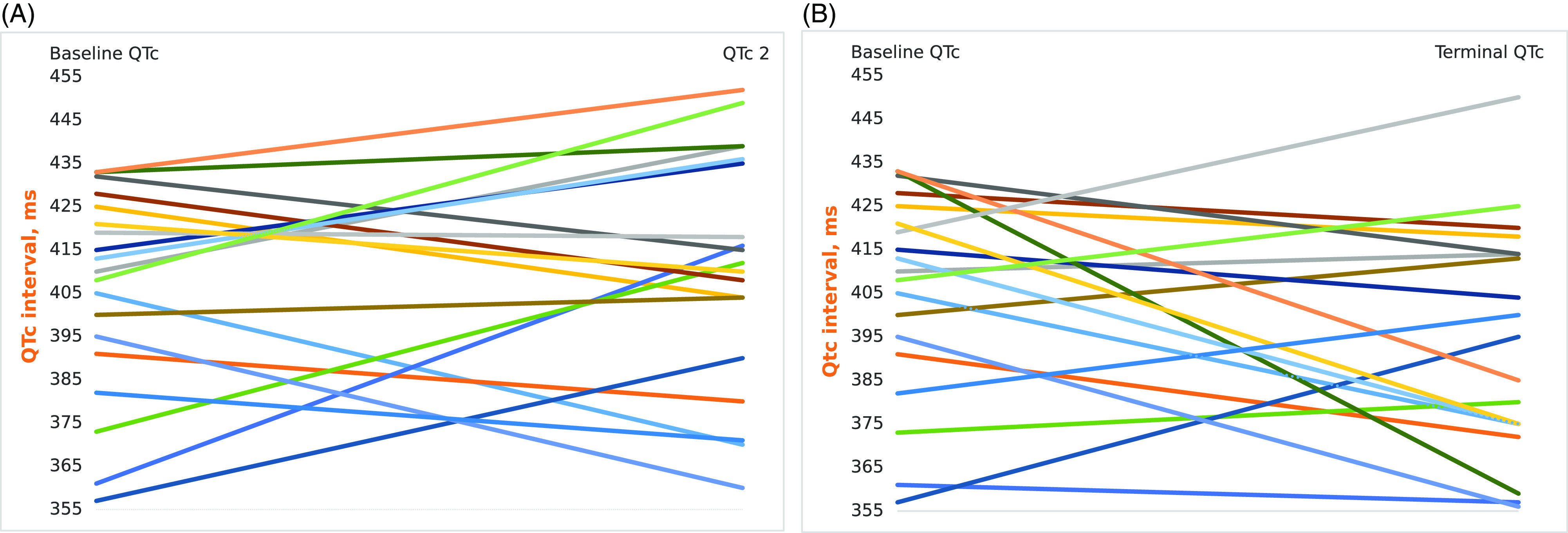
QTc values of the individuals on hydroxychloroquine and azithromycin at the baseline and one day after the treatment (A), baseline and at the termination of the treatment (B) (QTc = corrected QT interval; QTc 2 = QTc one day after the treatment).

Although the baseline corrected QT was shorter in patients receiving combined azithromycin and hydroxychloroquine than the patients receiving only hydroxychloroquine (median 410 [357–433] milliseconds versus 440 [395–486] milliseconds) neither the patients receiving hydroxychloroquine monotherapy, nor the ones receiving hydroxychloroquine plus azithromycin developed prolonged corrected QT of 500 milliseconds or more during or after the termination of medications. ΔQTc was under 60 milliseconds for all of the patients. We did not find a relation between the patients receiving concomitant azithromycin compared with those taking hydroxychloroquine alone in terms of Δ corrected QT just after the initiation of the therapy as well as at the termination of it. Data of corrected QT of individuals receiving hydroxychloroquine plus azithromycin at baseline and one day after use of drugs as well as baseline and at the termination of the treatment are shown in the Figure 1.

## Discussion

Hydroxychloroquine is structurally and mechanically similar to the class The Vaughan Williams classification of an antiarrythmic agent (IA) antiarrhythmic quinidine, which inhibits voltage-gated sodium and potassium channels, prolonging the QT interval and increasing the risk of Torsades de pointes and sudden cardiac death.^5^ Azithromycin also has been implicated in corrected QT prolongation and proarrhythmic events.^7^ In a systematic review studying the arrhythmogenic cardiotoxicity of the quinoline and structurally related antimalarial drugs, in total, 177 articles enrolling a total of 39,960 participants were included. There were no sudden deaths attributed to cardiac arrhythmias recorded in the > 35,000 individuals who received the quinoline and structurally related anti-malarials in the 177 clinical trials included. Despite vast use of chloroquine over the last six decades, only 1076 participants in 17 studies of chloroquine underwent electrocardiogram investigation. Total number of studies which included children were 6.^8^ Chloroquine is the most widely used anti-malarial drug in history. It has a terminal elimination half-life of one month and an annual consumption of hundreds of tons for over 50 years, so it may be the drug to which humans have been exposed to most.^9^ Despite producing consistent QT prolongation, the only case reports of Torsades de pointes and sudden death have been for its use for non-malaria indications such as systemic lupus erythematosus or rheumatoid arthritis, where high doses are used for much longer than in malaria treatment, or in overdose.^5^ Combined use of hydroxychloroquine and azithromycin was associated with a higher risk of dose-dependent QT prolongation than hydroxychroloquine monotherapy. The maximal mean prolongation with the co administration of azithromycin 500, 1000, and 1500 mg was 5 milliseconds (95% upper confidence interval: 10 milliseconds), 7 milliseconds (95% upper confidence interval: 12 ms), and 9 milliseconds (95% upper confidence interval: 14 milliseconds), respectively.^10^ There are also conflicting results regarding azithromycin and QTc interval. Dunker et al. reported that they had observed no significant change in QTc interval monitoring with baseline or follow-up electrocardiogram monitoring among patients receiving azithromycin.^11^ Two hundred one patients were treated for coronavirus disease 2019 with chloroquine/hydroxychloroquine on a study conducted on March 2020. The study revealed that in the largest reported cohort of coronavirus disease 2019 patients to date treated with chloroquine/hydroxychloroquine (plus minus) azithromycin, no instances of Torsades de pointes or arrhythmogenic death were reported. Although use of these medications resulted in QT prolongation, clinicians seldom needed to discontinue therapy.^12^ However, on the contrary, a study published in May 2020 showed that patients who received hydroxychloroquine for the treatment of pneumonia associated with coronavirus disease 2019 were at high risk of corrected QT prolongation, and concurrent treatment with azithromycin was associated with greater changes in corrected QT. Among 90 patients given hydroxychloroquine, 53 received concomitant azithromycin. Seven patients (19%) who received hydroxychloroquine monotherapy developed prolonged corrected QT of 500 milliseconds or more, and three patients (3%) had a change in corrected QT of 60 milliseconds or more. Of those who received concomitant azithromycin, 11 of 53 (21%) had prolonged corrected QT of 500 milliseconds or more and 7 of 53 (13 %) had a change in corrected QT of 60 milliseconds or more. The likelihood of prolonged corrected QT was greater in those who received concomitant loop diuretics. Ten patients had hydroxychloroquine discontinued early because of potential adverse drug events, including intractable nausea, hypoglycaemia, and one case of torsades de pointes.^13^ Yet another study of May 2020 also supports this hypothesis. It is a retrospective study of 251 patients from two centres. Study conducted by Chorine et al. showed corrected QT prolongation in parallel with increasing drug exposure and incompletely shortens after its completion. Extreme new corrected QT prolongation to > 500 milliseconds, a known marker of high risk for Torsades de pointes had developed in 23% of patients. One patient developed polymorphic ventricular tachycardia suspected as Torsades de pointes, requiring emergent cardioversion. Seven patients required premature termination of therapy.^14^ Although most of these data are based on adult studies, accumulated data show conflicting results on cardiac rhythm as well as corrected QT. Our study partially supports this fact as none of our patients showed neither corrected QT prolongation nor other cardiac adverse effects. However, it is evident that the patients in our study are clinically different from patients who are critically ill and receiving multiple corrected QT prolonging medications with extended half-lives, which augment cardio toxic risks. They also did not have hypokalaemia, hypocalcaemia or hypomagnesaemia which may lead to QT interval prolongation. Although our patients had findings of pulmonary involvement none of them had respiratory failure needing mechanical ventilation support. Moreover, it is imperative to stress that our study population is small and a relatively sterile group as only two of them had asthma which did not intervene with the progression of the disease. They were treated in our inpatient clinic not in a paediatric ICU where they would be posed to electrolyte imbalances, arrhythmia inducing myocardial damages and different other conditions leading to corrected QT prolongation. Another important fact is that both of the medications were given for a limited time of five days where as systemic lupus erythematosus, rheumatoid arthritis, or malaria needs prolonged treatment. Finally, extreme caution should be exercised while treating patients with inherited and acquired long QT syndrome in the setting of the coronavirus disease 2019 pandemic. QT intervals should be monitored at baseline and at 4 hours after the administration of hydroxychloroquine and/or azithromycin. All other non-critical QT prolonging agents must be discontinued. Serum potassium should be monitored and optimised daily. If QTc increases by >60 milliseconds or absolute QTc >500 milliseconds (or >530–550 milliseconds if QRS > 120 milliseconds), intensified monitoring, raising potassium levels, and/or discontinuation or dose reduction of QT prolonging drugs should be considered re-evaluating the risk/benefit of ongoing therapy.^15,16^
